# Supplementary material for: Fungus-Based MnO/Porous Carbon Nanohybrid as Efficient Laccase Mimic for Oxygen Reduction Catalysis and Hydroquinone Detection
Source: Nanomaterials (Basel). 2022 May 8;12(9):1596. doi: 10.3390/nano12091596 (PMC9103193; doi:10.3390/nano12091596)
Supplement: Supplementary file 1 [file nanomaterials-12-01596-s001.zip › nanomaterials-1677907-supplementary.pdf]

Supporting information

Fungus Based MnO/Porous Carbon Nanohybrid as Efficient Laccase Mimic for Oxygen Reduction Catalysis and Hydroquinone Detection

Haoran Ge, Hailong Zhang*
School of Electrical and Automation Engineering, Nanjing Normal University, Nanjing 210023, China

* Email: zhanghl@njnu.edu.cn

Electrochemical Test

To prepare the working electrode, 3.5 mg of MnO/PC nanohybrid mixed with 1.5 mg Cabot Vulcan XC-72 carbon (Vc-72) were dispersed in a solution containing 500 μL of mixed solution (2-propanol mixed with water (V_{C3H8O}:V_{H2O} = 1:1) and 0.5 wt % Nafion solution). The suspension was ultrasonically dispersed to form a homogeneous ink. After that, the ink was pipetted onto the GC electrode and then was naturally drying to form a thin catalyst layer on the GC electrode. The tests were conducted on a computer-controlled potentionstat/galvanostat workstation at room temperature. The supporting electrolyte was 0.1 M KOH aqueous solution, which was purged with N_2 or O_2 (purity 99.995%) for at least 30 min prior to testing and maintained under N_2 or O_2 atmosphere during the test. Cyclic voltammograms were recorded from 0.2 to -0.8 V versus Ag/AgCl in N_2 (or) O_2-staurated 0.1 M KOH electrolyte solutions with a scan rate of 20 mV s^{-1}. All potentials were reported with reference to the reversible hydrogen electrode (RHE) potential scale. In 0.1 mol L^{-1} KOH solution, the potential of Ag/AgCl was calibrated as +0.965 V with respect to RHE.

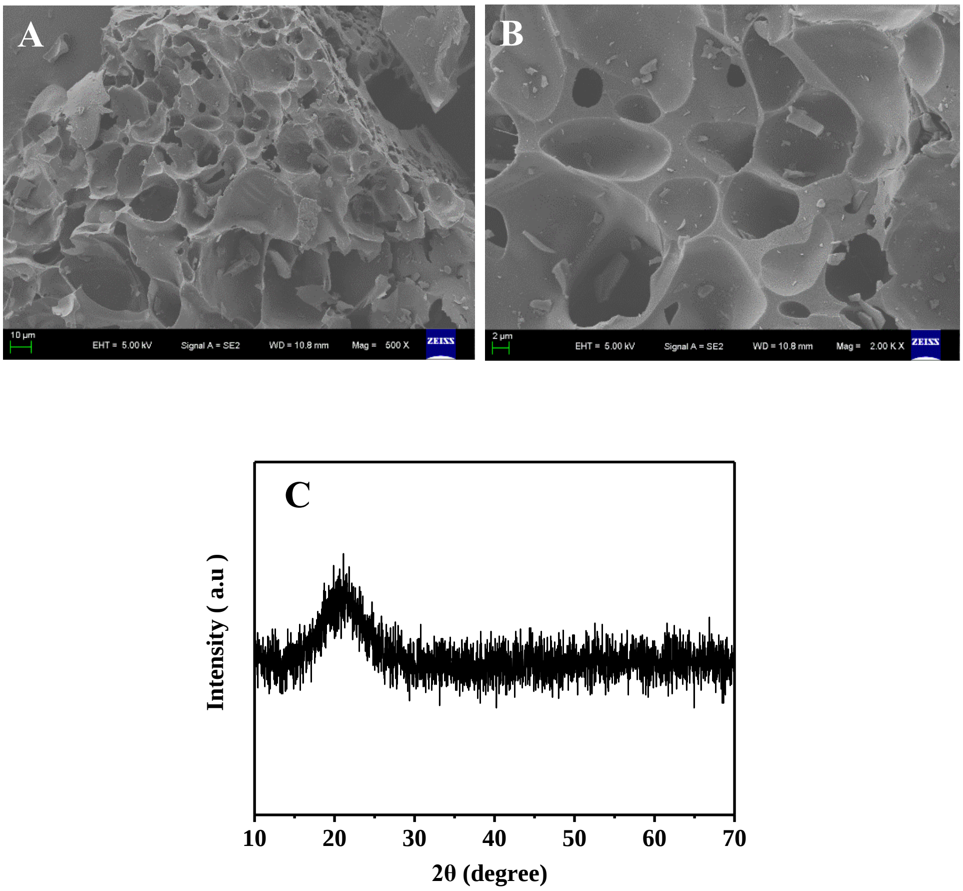

Figure S1. (A, B) SEM images of porous carbon with different magnifications. (C) XRD patterns of porous carbon.

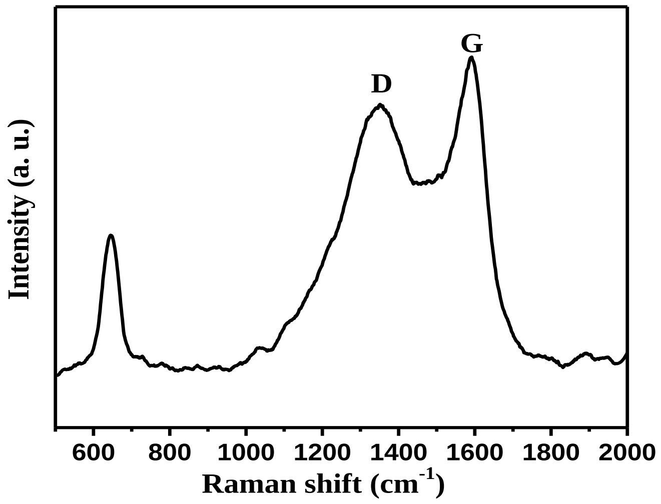

Figure S2. Raman spectra of MnO/PC nanohybrid.

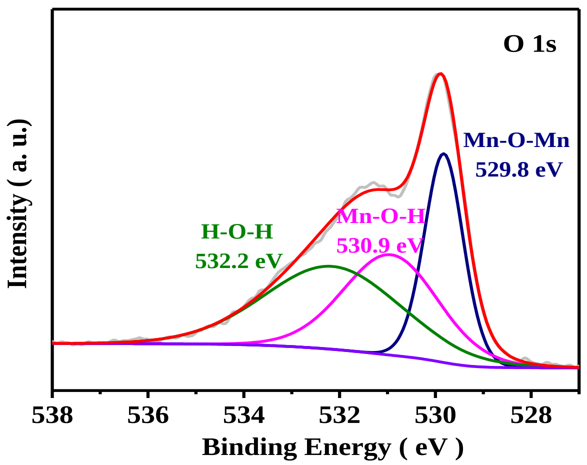

Figure S3. High-resolution O1s XPS spectra of MnO/PC nanohybrid.

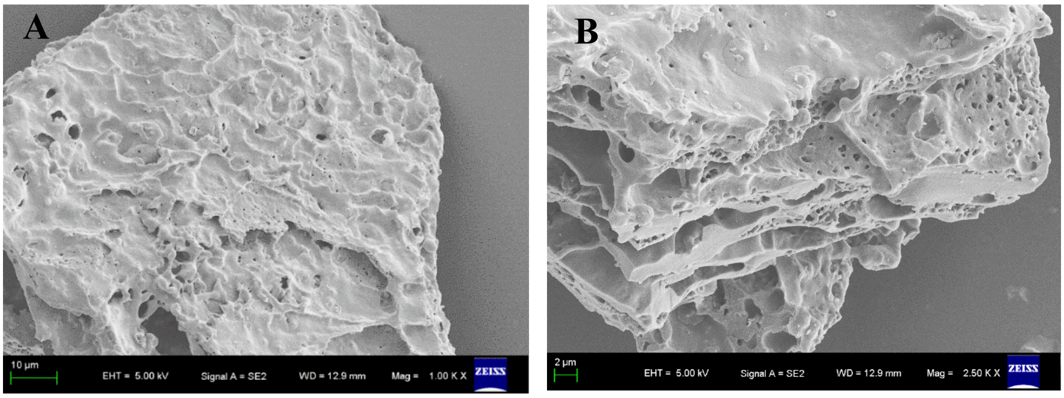

Figure S4. SEM images MnO/PC nanohybrid obtained at 700 °C with different magnifications.

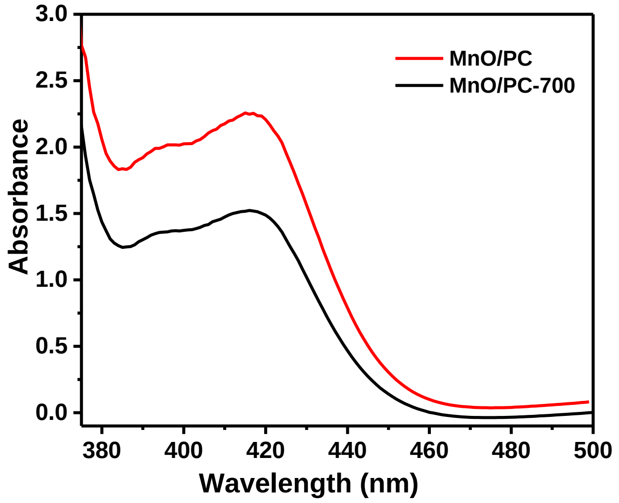

Figure S5. Typical UV-vis spectra of the oxidation of ABTS by MnO/PC nanohybrid obtained at 700 and 800 °C.

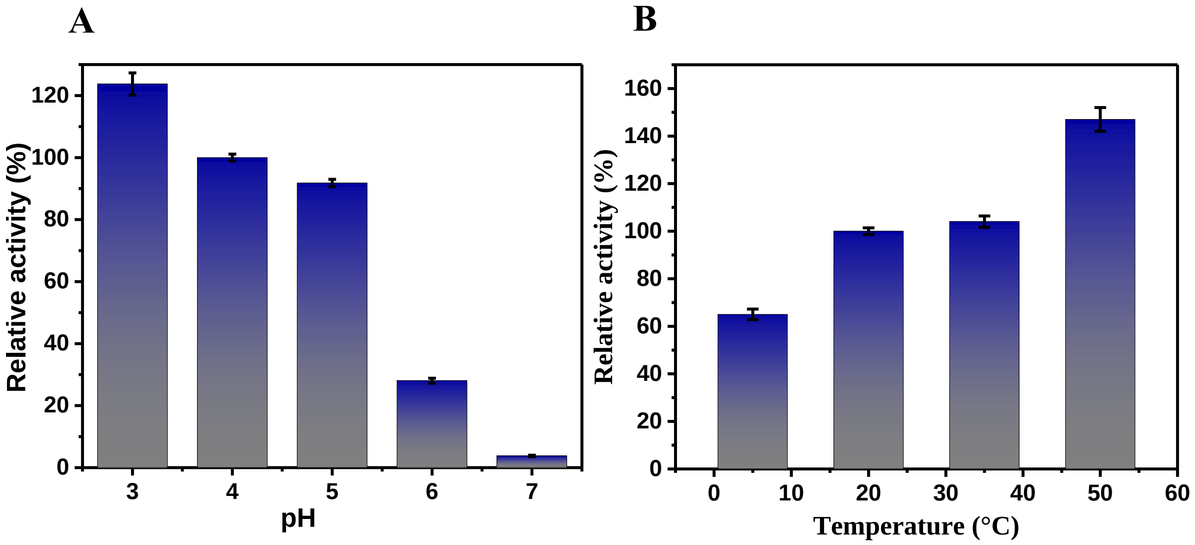

Figure S6. The influence of pH (A) and temperature (B) on the relative activity of the MnO/PC nanohybrid.

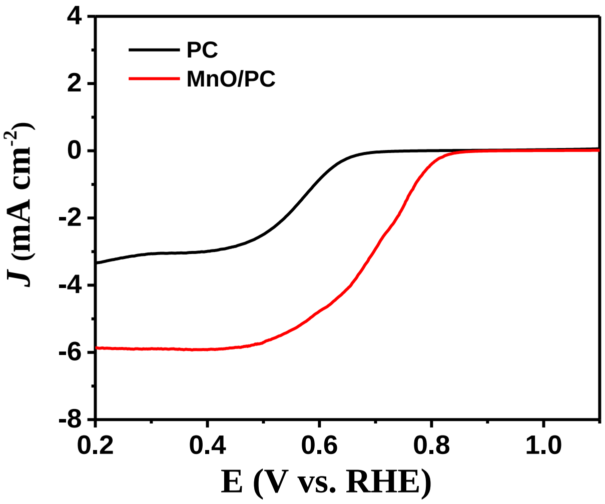

Figure S7. LSV curves of PC and MnO/PC nanohybrid at 1600 rpm in O_2-saturated 0.1 M KOH.

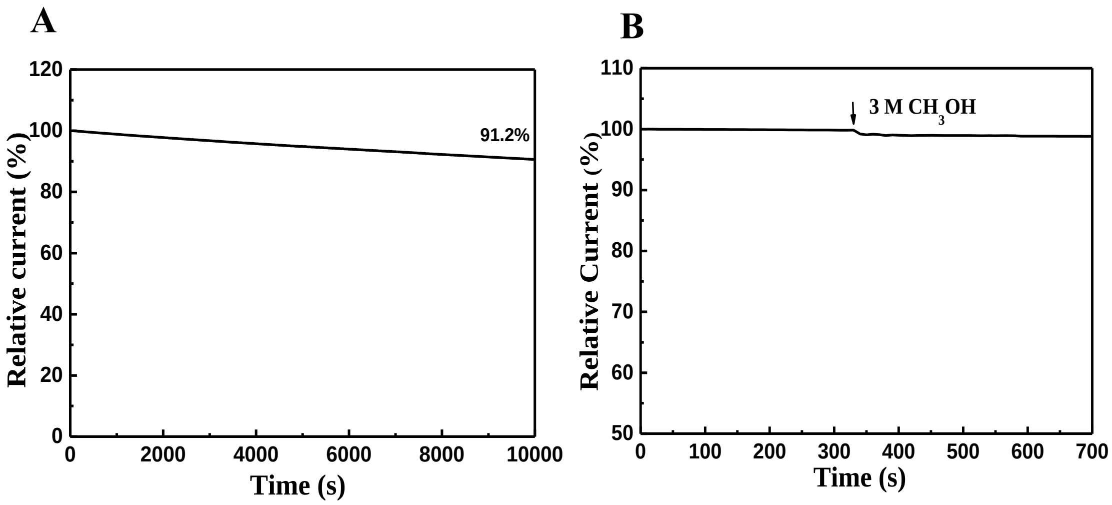

Figure S8. (A) Chronoamperometric curves of MnO/PC nanohybrid and Pt/C in O_2-saturated 0.1M KOH with a constant potential 0.65 V (vs. RHE). (B) Chronoamperometric curves of MnO/PC nanohybrid by adding of 3 M methanol after 330 s in O_2-saturated 0.1 M KOH with a constant potential 0.65 V (vs. RHE).

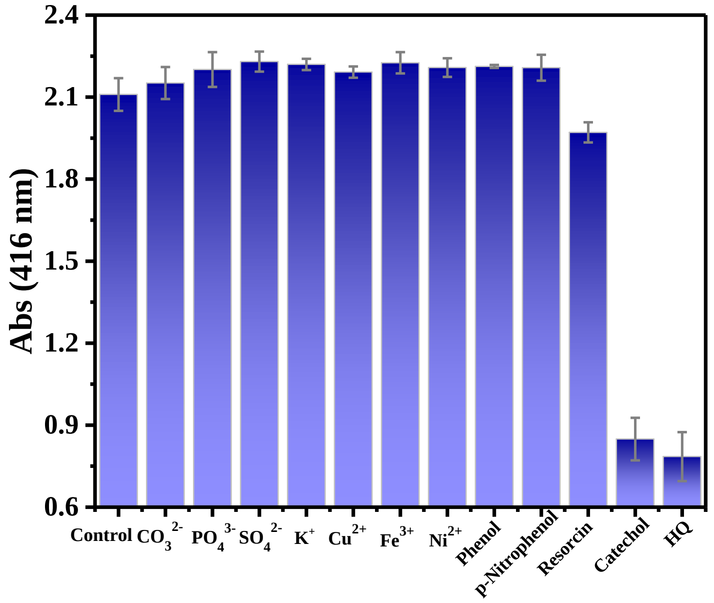

Figure S9. Effect of different phenols and ions on HQ sensing with adding the equal amount of phenols and 10-fold other compounds.

Table S1. Comparison of the ORR electrochemical performances of MnO/PC nanohybrid with the reported catalysts and Pt/C.

Catalyst	E_{onset} (V *vs*. RHE)	Electron transfer number	Limiting current density (mA cm^{-2})	Reference
MnO	--	2.18	1.1	1
MnO/NG	0.89	3.70	4.17	2
MnO	0.70	--	1.1	3
3D-N-RGO/MnO	0.83	3.03	1.62	3
MnO	--	2.94-3.14	1.0	4
MnO/RGO	--	3.98-4.02	4.72	4
MnO/NC-2	0.987	4	5.4	5
CNTs@MnO	-	3.8–4.0	1.6	6
CNTs@MnO_x	-	3.2–3.4	1.4	6
Laccase-mimicking Cu−DPA	0.78	3.7	--	7
MnO_x/C	--	3.54	--	8
Pt/C-20%	0.93	3.9	5.96	This work
MnO/PC nanohybrid	0.83	3.9	5.87	This work

Table S2. Determination of HQ in real sample using the present colorimetrical method (n= 3).

Sample	Added (μM)	Found (μM)	Recovery (%)	RSD (n = 3, %)
Tap water	15.0	15.0	100.0	4.0
	30.0	28.8	96.0	3.0
Lake water	15.0	14.5	96.7	2.7
	30.0	31.9	106.3	4.6

References

1. Wu, X.; Gao, X.; Xu, L.; Huang, T.; Yu J.; Wen, C.; Chen, Z.; Han, J. Mn_2O_3 doping induced the improvement of catalytic performance for oxygen reduction of MnO. *Int. J. Hydrogen Energy* **2016**, 41, 16087-16093.
2. Arunchander, A.; Vivekanantha, M.; Peeraa, S. G.; Sahu, A. K. MnO–nitrogen doped graphene as a durable non-precious hybrid catalyst for the oxygen reduction reaction in anion exchange membrane fuel cells. *RSC Adv.* **2016**, 6, 95590–95600.
3. Chen, R.; Yan, J.; Liu, Y.; Li, J. Three-dimensional nitrogen-Doped graphene/MnO nanoparticle hybrids as a high-performance catalyst for oxygen reduction reaction. *J. Phys. Chem. C*. **2015**, 119, 8032-8037.
4. Wu, Q.; Jiang, M.; Zhang, X.; Cai, J.; Lin, S. A novel octahedral MnO/RGO composite prepared by thermal decomposition as a noble-metal free electrocatalyst for ORR. *Mater Sci.*, **2017**, 52, 6656-6669.
5. Ding, J.; Ji, S.; Wang, H.; Brett, D. J. L.; Pollet, B. J.; Wang, B. G. MnO/N-doped mesoporous carbon as advanced oxygen reduction reaction electrocatalyst for zinc–air batteries. *Chem. Eur. J.* **2019**, 25, 2868 – 2876.
6. Luo, W.; Chou, S.; Wang, J.; Zhai, Y.; Liu, H. A facile approach to synthesize stable CNTs@MnO electrocatalyst for high energy lithium oxygen batteries. *Scientific Reports* 2015, 5, 8012.
7. Edmund, C. M. T.; David, S.; Danielle, L. G.; Thomas, B. R.; Andrew, A. G. Multicopper models for the laccase active site: effect of nuclearity on electrocatalytic oxygen reduction. *Inorg. Chem.* **2014**, 53, 8505−8516.
8. Roche, I.; Chaînet, E.; Chatenet, M.; Vondrák, J. Carbon-supported manganese oxide nanoparticles as electrocatalysts for the oxygen reduction reaction (ORR) in alkaline medium: physical characterizations and ORR mechanism. *J. Phys. Chem. C* **2007**, 111, 1434-1443.
